# *Escherichia coli* O157:H7 in Feral Swine near Spinach Fields and Cattle, Central California Coast^1^

**DOI:** 10.3201/eid1312.070763

**Published:** 2007-12

**Authors:** Michele T. Jay, Michael Cooley, Diana Carychao, Gerald W. Wiscomb, Richard A. Sweitzer, Leta Crawford-Miksza, Jeff A. Farrar, David K. Lau, Janice O’Connell, Anne Millington, Roderick V. Asmundson, Edward R. Atwill, Robert E. Mandrell

**Affiliations:** * California Department of Public Health, Richmond, California, USA; †University of California, Davis, California, USA; ‡ US Department of Agriculture, Albany, California, USA; § US Department of Agriculture, Sacramento, California, USA; ¶ University of North Dakota, Grand Forks, North Dakota, USA; # California Department of Public Health, Sacramento, California, USA; ** US Food and Drug Administration, Alameda, California, USA

**Keywords:** Escherichia coli O157, Sus scrofa, wild boar, California, DNA typing, population density, spinach, swine, dispatch

## Abstract

We investigated involvement of feral swine in contamination of agricultural fields and surface waterways with *Escherichia coli* O157:H7 after a nationwide outbreak traced to bagged spinach from California. Isolates from feral swine, cattle, surface water, sediment, and soil at 1 ranch were matched to the outbreak strain.

Recent experimental and epidemiologic studies suggest that domestic pigs are biologically competent hosts and a potential reservoir of *Escherichia coli* O157:H7 (*1**,**2*). Cattle are considered the primary reservoir of *E*. *coli* O157, but fecal shedding by other domestic livestock and wildlife has been described (*3**,**4*). *E*. *coli* O157 was isolated from a wild boar in Sweden, but there is limited information on its occurrence in feral swine in the United States (*5*). We report findings from an environmental and laboratory investigation after a nationwide spinach-associated outbreak of *E*. *coli* O157 in which the outbreak strain was isolated from feral swine and other environmental samples.

## The Study

In September 2006, an outbreak of *E*. *coli* O157 was linked to consumption of fresh, bagged, baby spinach, with 26 states and Canada reporting 205 cases of illness and 3 deaths (*6*). Contaminated product was traced to 1 production date (August 15, 2006) at 1 processing plant and fields located on 4 ranches on the central California coast (*7*). The outbreak strain was isolated initially from cattle feces collected on September 27, 2006, ≈1 mile from an implicated spinach field on a ranch (ranch A) where numerous free-roaming feral swine were observed. We investigated potential involvement of feral swine in *E*. *coli* O157 contamination of spinach fields and surface waterways.

**Figure 1:**
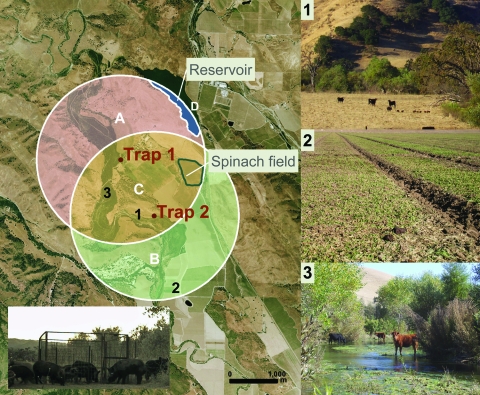
Left: aerial (2 m) photograph of ranch A showing overlapping circular buffer regions around feral swine trap 1 and trap 2 (San Benito Crop Year 2006; Image Trader, Flagstaff, AZ). The radius for the buffer (1.8 km) is the circumference of the mean home range for feral swine in mainland California (*8*). Estimated density = 4.6 swine/km^2^ and total area = (A + B + C) – D = 14.8 km^2^. Areas A, B, and C, combined with counts of individual feral swine from October through November 2006, were used to calculate the average population density. Bottom left: digital infrared photograph of feral swine at trap 1. Right: potential risk factors for *Escherichia coli* O157:H7 contamination of spinach at ranch A: 1) Feral sow and piglets sharing rangeland with cattle; 2) feral swine feces, tracks, and rooting in a neighboring spinach field; 3) cattle in surface water.

Feral swine were live-captured in traps or hunted and humanely killed during October–November 2006. Two feral swine corral traps were placed 1.4 km apart, and 1.7 km (trap 1) and 1.2 km (trap 2), respectively, from the implicated spinach field (Figure 1). Photographs from digital infrared remote-sensing cameras (Recon Outdoors, Huntsville, AL, USA) were used in combination with sightings and live-capture to ascertain the minimum number of individual feral swine present on the ranch (*8*). The average population density was calculated on the basis of an estimate of the area sampled by both traps and the estimated mean home range (1.8 km) for feral swine in mainland California by using ArcView version 9.2 (Environmental Systems Research Institute, Redlands, CA, USA) (*8*).

Colonic fecal samples were collected from 40 feral swine (31 live-captured, 9 hunted); buccal swabs, rectal-anal swabs, and tonsils were analyzed from a subset of 8 animals (Table 1). Additionally, feces from domestic animals (cattle, dog, goat, horse, sheep) and wildlife (bird, coyote, deer, feral swine), surface water and sediment, soil, and well/irrigation water were analyzed. *E*. *coli* O157 was cultured by using an extended enrichment–immunomagnetic separation protocol (*9*,*10*). PCR analysis was used to confirm the presence of *E*. *coli* O157 and virulence factors (*9**,**10*). Genotypes of isolates from environmental samples were compared by using 10-loci multilocus variable number tandem repeat analysis (MLVA) and pulsed-field gel electrophoresis (PFGE) after digestion with *Xba*I and *Bln*I by using the PulseNet protocol (*10**–**13*).

**Table 1:** *Escherichia coli* O157:H7 isolated from environmental samples collected at ranch A, California, September–November 2006

Sample type	No. tested	No. positive (%)	No. matches*
Cattle feces	77	26 (33.8)	15
Cattle water trough	10	0	NA
Compost (chicken pellets)†	1	0	NA
Feral swine			
Necropsy	40	2 (5)	2
Buccal swab	8	0	NA
Colonic feces	40	2 (5)	2
Rectal-anal swab	8	0	NA
Tonsil	8	0	NA
Feces from ground	47	11 (23.4)	6
Subtotal	87	13 (14.9)	8
Other animal specimens‡	26	0	NA
Surface water§	79	3 (3.8)	2
Soil/sediment	37	3 (8.1)	3
Well/irrigation water¶	18	0	NA
Total	335	45 (13.4)	28

*No. samples indistinguishable from the major spinach-related outbreak strain by pulsed-field gel electrophoresis (*Xba*I-*Bln*I PulseNet profile EXHX01.0124-EXHA26.0015). NA, not applicable. †Commercial, heat-treated chicken manure. ‡Included feces from coyote (n = 1), deer (n = 4), dog (n = 1), horse (n = 2), sheep/goat (n = 3, composite), waterfowl (n = 2), unknown species (n = 11), and owl (n = 2). §Surface water (rivers, streams, ponds) was sampled by collection of 100-mL grab samples or placement of a modified Moore swab for 4–5 d. ¶Well water was sampled from 3 wells or sprinkler heads by collection of 100-mL or 1,000-mL grab samples or by concentration of 40,000 mL to 500 mL by using ultrafiltration (*7*).

**Figure 2:**
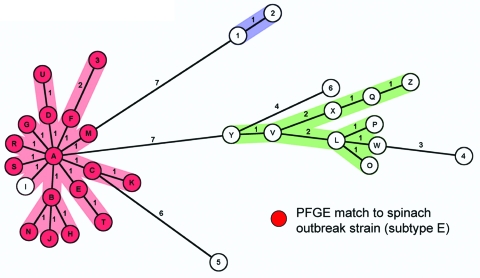
Minimum spanning tree analysis of multilocus variable number tandem repeat analysis (MLVA) data of 76 *Escherichia coli* O157:H7 strains typed from 47 samples compared with the spinach-related outbreak strain (subtype E). A categorical coefficient and the BURST priority rule of the highest number of single-locus changes were used for the clustering (Bionumerics software version 4.601, Applied Maths, Austin, TX, USA). Circles representing unique MLVA types are designated by an alphanumeric value (Table 2). Numbers between circles represent summed tandem-repeat differences between MLVA types (*10*). The shaded areas (red, green, and blue) denote genetically related clusters with MLVA differences <3. Red circles indicate types comprising isolates that were indistinguishable from the spinach-related outbreak strain (subtype E) by pulsed-field gel electrophoresis (PFGE).

*E*. *coli* O157 was cultured from 45 (13.4%) of 335 samples, including cattle and feral swine feces, feral swine colonic feces from necropsy, surface water and sediment, and pasture soil (Table 1). The *eaeA*, *hlyA*, and *stx2* genes were present in all strains, and the *stx1* gene was found in only 1 sample (subtype 5; Table 2, Figure 2). Isolates from 28 environmental samples at ranch A were indistinguishable from the major spinach-related outbreak strain by PFGE (Table 1). In contrast, *E*. *coli* O157 isolates from 3 other ranches implicated by traceback did not match the outbreak strain. Molecular typing by MLVA provided higher resolution discrimination between environmental strains (Figure 2). Three major MLVA clusters from ranch A and the surrounding watershed were identified. The cluster containing the outbreak strain (subtype E) is shown in Figure 2, and 16 other highly related subtypes were indistinguishable by PFGE (Table 2).

**Table 2:** Unique alphanumeric MLVA types of *Escherichia coli* O157:H7 isolated from environmental samples collected at ranch A and an upstream watershed, California, September–November 2006*

Sample type	No. samples	No. isolates	MLVA type
Reference (human stool, bagged spinach)	NA	NA	**E**
Cattle feces	26	34	**A**, **C**, **E**, **F**, I, **J**, L, **M**, P, Q, **R**, **S**, **T**, W, X, Z
Feral swine feces	11	14	**A**, **B**, **C**, **E**, L, O, P, X, 5, 6
Feral swine colonic feces (necropsy)	2	10	**A**, **C**, **D**, **G**, **H**, **K**, L, **U**, V, Y
Sediment (river)	2	8	**A**, **C**, L, **M**, **N**, W, **3**
Soil (cattle pasture)	1	1	**A**
Surface water	3	6	**A**, **C**, L, P, 4
Surface water Moore swab†	2	3	1, 2

*MLVA, multilocus variable number tandem repeat analysis; NA, not applicable. Samples indistinguishable from the major spinach-related outbreak strain by pulsed-field gel electrophoresis (*Xba*I-*Bln*I PulseNet profile EXHX01.0124-EXHA26.0015) are shown in **boldface**. †Isolates collected from surface water (river) ≈32 km upstream of ranch A.

Ranch A is located in the central coast foothills of San Benito County, where the dominant habitat is coastal oak woodland interspersed with dense riparian vegetation near seasonal waterways (Figure 1). Approximately 2,000 range cattle were grazed on the ranch. Spinach and other leafy green vegetables were grown on a leased portion of the property that was separated from cattle pastures by wire mesh fence. Well water was used for irrigation. No evidence of cattle manure–based fertilizer application, runoff from cattle pastures, or flooding from surface waterways (based on topography) onto the implicated spinach field was found during the investigation (*7*).

Feral swine were the most abundant wildlife observed on ranch A, and evidence of intrusion, including tracks, rooting, or feces in crop fields and adjacent vineyards, was documented (Figure 1). Birds, black-tailed deer, cottontail rabbits, coyotes, and ground squirrels also were observed, but the population density of these species appeared lower, and their activity was confined mostly to rangeland areas according to visual observations. Swine visited the traps almost continuously from dusk until dawn with peak activity between 5:00 pm and midnight. An average of 3.6 swine/trap/night were live-captured. The estimated population density was 4.6 swine/km^2^ (95% confidence interval [CI] 3.8–5.9), and the actual number of feral swine on ranch A was estimated to be 149 animals (95% CI 124–192) (Figure 1). Feral swine used livestock rangelands and gained access to adjacent crop fields through gaps formed at the base of the fence by erosion and rooting. Cattle and feral swine had access to and congregated at surface waterways on the ranch (Figure 1).

## Conclusions

We describe the first, to our knowledge, isolation of *E*. *coli* O157 from feral swine in the United States. The percentage of specimens positive for *E*. *coli* O157 among feral swine (14.9%) and cattle (33.8%) and the density (4.6 swine/km^2^) were high compared with results of previous ecologic studies (Table 1) (*2**–**5**,**8**,**14**,**15*). Molecular typing of isolates by PFGE and MLVA showed possible dissemination and persistence of the outbreak strain in multiple environmental samples as long as 3 months after the outbreak (Tables 1, 2). MLVA is more reproducible than PFGE and better at discriminating between closely related *E*. *coli* O157 isolates (*10*,*12*,*13*). Recovery of related *E*. *coli* O157 subtypes by both methods suggested swine-to-swine transmission, interspecies transmission between cattle and swine, or a common source of exposure such as water or soil (Table 2, Figure 2).

Mechanisms of in-field contamination of leafy greens for this and previous outbreaks remain unclear, but hypotheses have emerged. A relatively high density of feral swine near cattle and spinach fields could represent a risk factor for *E*. *coli* O157 contamination. Wildlife may be sentinels for *E*. *coli* O157 in the produce production environment, or they may be vectors involved in the contamination of plants directly by fecal deposition or indirectly by fecal contamination of surface waterways or soil. Notably, baby spinach is harvested with a lawn mower–like machine that could pick up fecal deposits in the field and thereby contaminate large volumes of product during processing. Fecal loading of surface waterways by livestock and wildlife with subsequent contamination of wells used for irrigation represents another possible route of transmission to plants in the field. Although *E*. *coli* O157 was not detected in irrigation water, older agriculture wells at ranch A appeared vulnerable to contamination by surface water (R. Gelting, pers. comm.). Unrecognized environmental and management practices during preharvest and postharvest processing also could have contributed to amplification and dissemination of *E*. *coli* O157 in raw spinach.

In summary, *E*. *coli* O157 contamination of spinach and other leafy greens is likely a multifactorial process. Additional research is needed to develop and implement effective risk assessment and management practices. For example, studies are needed to determine colonization potential of and levels of fecal shedding by feral swine, and the importance of interspecies transmission to other vertebrate or invertebrate (e.g., flies) populations near agricultural fields.
